# Construct α-FeOOH-Reduced Graphene Oxide Aerogel as a Carrier for Glucose Oxidase Electrode

**DOI:** 10.3390/membranes12050447

**Published:** 2022-04-21

**Authors:** Yue Yao, Changyu Hou, Xin Zhang

**Affiliations:** 1 College of Chemistry and Materials Engineering, Anhui Science and Technology University, Bengbu 233030, China; h202986627@163.com; 2 Department of Chemistry, College of Science, Shantou University, Shantou 515063, China; xzhang@stu.edu.cn

**Keywords:** α-FeOOH, enzyme electrode, electrochemical catalysis, carrier

## Abstract

A promising α-FeOOH-reduced graphene oxide aerogel (FeOOH-GA) has been prepared for the assembly of an enzyme electrode. The α-FeOOH-reduced graphene oxide aerogel was characterized by X-ray powder diffraction, field emission scanning electron microscopy, transmission electron microscopy, Fourier transform infrared spectroscopy, Raman, energy-dispersive X-ray spectroscopy, and X-ray photoelectron spectroscopy. The results reveal that graphene oxide is reduced by Fe^2+^ ion and α-FeOOH nanorods anchored on the reduced graphene oxide sheet through the Fe-O-C bond. Analyses using scanning electron microscopy and the Brunauer–Emmett–Teller method show that FeOOH-GA displays a various and interconnected pore structure. The FeOOH-GA was used as a support material on the glass carbon electrode (GCE) for glucose oxidase (GOD). Electrochemistry properties and bioelectrocatalytic activities of Nafion/GOD/FeOOH-GA/GCE were achieved from cyclic voltammetry and electrochemical impedance spectroscopy. The results show that Nafion/GOD/FeOOH-GA/GCE maintains outstanding catalytic activity and electrochemical properties. The FeOOH-GA could immobilize GOD through the hydrophobicity of the reduced graphene oxide and hydroxide radical of α-FeOOH. Appropriate α-FeOOH and diversified pore structure are beneficial for electron transfer, enzyme electrode storage, and interfacial electron transfer rate. All results indicated that the α-FeOOH-reduced graphene oxide aerogel as a carrier could effectively immobilize the tested enzyme.

## 1. Introduction

Enzyme electrodes are important parts of enzyme biofuel cells (EBFC) due to the electrochemical reaction that takes place in them. Glucose oxidase (GOD) as a perfect oxidase-type enzyme is generally used in bioelectrochemistry research because of its outstanding catalytic activity, good stability, and wonderful selectivity for β-D-glucose [1,2].

The half-life period of free enzyme is only a few hours in solution. However, the lifetime of immobilized enzyme is greatly improved [3]. Therefore, there is an important problem in EBFC, namely how to choose support materials and immobilization strategies for enhancing the stability and maintaining the activity of enzyme electrodes [4]. As a result of its unique electronic structure, significant electrical conductivity, excellent biocompatibility, and easy functionalization, carbon material has become a focus in research concerning supporters for enzyme electrodes. Within the last two decades, various types of carbon materials used for loading enzymes have been published, such as carbon nanotube [5], carbon quantum dot [6], carbon nanoparticle [7], graphene [8], graphene oxide [9], and so on. Among them, graphene with large surface area has wide application prospects. Kang [10] et al. reported that GOD fixated on graphene/chitosan effectively catalyzed the oxidation reaction of glucose. Then, the electrode modified with GOD-graphene-chitosan was used as a biosensor for glucose sensing, exhibiting a high sensitivity and stability. However, the irreversible aggregation of graphene sheet produced surface area minification, which was deleterious for enzyme immobilization. The graphene aerogel and reduced graphene oxide aerogel with a plentifully diverse porous structure represent a three-dimensional carbon material [11]. In comparison with stacked two-dimensional graphene sheet, three-dimensional porous structures can adsorb a greater quantity of enzyme [12]. Moreover, a porous structure is not only advantageous for the adsorption of substance, but also for the entrance of solution, which provides a comfortable reaction condition for enzyme. Despite their many advantages, porous structures leak out enzymes in storage. Among the various inorganic nanoparticles, Fe-based oxides nanoparticles are investigated popularly as a consequence of their good biocompatibility, low cost, minimal toxicity, and so on [13,14,15]. The iron hydroxyl oxide (FeOOH) nano-material has a particular surface because of hydroxyl groups (OH-), which can interact with the enzyme instead of adding other function groups [16]. Goethite (α-FeOOH) not only has the advantages of Fe-based oxide nanoparticles, but also has hydroxyl groups (OH-) on the surface. Therefore, it is a novel strategy that α-FeOOH nanoparticles are designed to form a buffer region in the porous structure and fix enzymes. 

Herein, graphene oxide was reduced by Fe^2+^ ion to form a reduced graphene oxide aerogel. At the same time, α-FeOOH nanorods were obtained. The α-FeOOH-reduced graphene oxide aerogel (FeOOH-GA) was successfully prepared to decorate the glass carbon electrode (GCE). Glucose oxidase (GOD) was immobilized on modified electrodes utilizing the hydrophobicity of the reduced graphene oxide sheet and hydroxide radical of α-FeOOH. Nafion/GOD/FeOOH-GA/GCE displayed outstanding catalytic activity and electrochemical properties. The results indicated that FeOOH-GA as a carrier could effectively immobilize the tested enzyme.

## 2. Experimental Details

### 2.1. Materials and Apparatus

Glucose oxidase (GOD, ≥180 u mg^−1^, from Aspergillus niger, 160 kDa, EC 1.1.3.4) and N-Hydroxy succinimide (NHS) were obtained from Aladdin chemical Agents Co., Ltd., (Shanghai, China). 1-ethyl-3-(3-dimethyl aminopropyl) carbodiimide hydrochloride (EDC) was purchased from Adamas-beta Inc. (Shanghai, China). The Nafion 5 wt% and Graphite (natural, 325 mesh) were purchased from Dupont and XFNaNo Inc. (Nanjing, China), respectively. P_2_O_5_, KMnO_4_, H_2_O_2_ (30%), H_2_SO_4_ (95–98%), HCl (36–38%), and CH_3_CH_2_OH were purchased from Xilong Chemical Reagent Co. Ltd. (Shantou, China). β-D-glucose, FeSO_4_·7H_2_O, NaOH, KCl, Ferrocenecarboxylic acid (Fc), Na_2_HPO_4_, NaH_2_PO_4_, K_3_[Fe(CN)_6_], and N, N-Dimethylformamide (DMF) were purchased from Macklin Biochemical Co., Ltd. (Shanghai, China).

Phosphate buffer solution (PBS, 0.2 M) used for electrochemical studies was prepared by mixing Na_2_HPO_4_ and NaH_2_PO_4._ The pH of PBS was adjusted to 1 M NaOH solution. The PBS including β-D-glucose was prepared at least 24 h before the experiment for mutarotation and kept at 4 °C. Deionized water (the specific resistance > 18.0 MΩ) used in all experiments was purified using a Millipore-Q purification system.

### 2.2. Preparation of FeOOH-GA

Graphene oxide (GO) was synthesized from natural graphite powder by a modified Hummer’s method [17,18] (for experimental details, see Supplementary Information (SI)). GO aqueous dispersion was obtained via dialysing for one week. The preparation of FeOOH-GA was performed according to the previously reported method [19]. Hence, 10 mL of 2 mg mL^−1^ GO aqueous dispersion was mixed with a certain amount of FeSO_4_·7H_2_O (0.125 mmol, 0.25 mmol, 1 mmol) in a 20 mL sampler vial with magnetic stirring to form a stable suspension. Then, 5 μL ammonia water was added. The sealed sampler vial was placed in an oil bath for 10 h at 90 °C without stirring. Finally, the cylindrical hydrogel was taken out, dialyzed against deionized water for 48 h, and lyophilized for further use. According to a certain amount of iron, the products were named as FeOOH-GA _0.125_, FeOOH-GA _0.25_, and FeOOH-GA _1.0_, respectively.

For comparison, FeOOH modified reduced graphene oxide power (FeOOH-GP) was also prepared. Thus, 0.835 g (0.25 mmol × 12) FeSO_4_·7H_2_O was added to 240 mL of 1 mg mL^−1^ GO aqueous dispersion in a round-bottomed flask with stirring until completely dissolved. Then, 30 μL ammonia water was added. The round-bottomed flask was placed in an oil bath for 10 h at 90 °C under refluxing and magnetic stirring. After cooling to room temperature, the black product was filtered by vacuum filtration and washed with deionized water, then freeze-dried for further use. 

### 2.3. Preparation of Working Electrodes

The pretreatment of a glass carbon electrode (GCE, diameter = 4 mm): A GCE was sequentially polished using a slurry of 0.3 μm and 0.05 μm alumina power, then successively washed in deionized water, ethanol, and deionized water under ultrasonication for 1.5 min, respectively, and dried by high purity N_2_. The electrode was examined with a typical three-electrode configuration in 5 mM K_3_[Fe(CN)_6_] + 0.1 M KCl by cyclic voltammetry (CV) under 50 mv s^−1^ scanning speed from −0.2 to 0.6 V.

Preparation of working Electrodes: 1 mg FeOOH-GA or FeOOH-GP was ultrasonically dispersed in 1 mL DMF to give a black suspension. Next, 10 µL of suspension was loaded on a usable GCE and dried at 60 °C to form FeOOH-GA/GCE or FeOOH-GP/GCE. After cooling to room temperature, 10 μL of 10 mg mL^−1^ GOD solution (0.2 M PBS pH = 7 including GOD) was loaded on FeOOH-GA/GCE or FeOOH-GP/GCE, stored for 90 min at room temperature, then dropped 10 μL of 0.2 M PBS including 36 mM EDC/17 mM NHS (pH = 5) and stored for 2 h at room temperature. Finally, 10 μL of Nafion solution (0.5 wt%) was added on the above GCE and dried at 4 °C overnight. Nafion/GOD/FeOOH-GA _0.125_/GCE, Nafion/GOD/FeOOH-GA _0.25_/GCE, Nafion/GOD/FeOOH-GA _1.0_/GCE and Nafion/GOD/FeOOH-GP/GCE were obtained and used as working Electrodes for further use. All prepared enzyme electrodes were stored at 4 °C when not in use.

For comparison, Nafion/FeOOH-GA _0.125_/GCE, Nafion/FeOOH-GA _0.25_/GCE, Nafion/FeOOH-GA _1.0_/GCE, and Nafion/FeOOH-GP/GCE were also prepared. 

### 2.4. Electrochemical Measurements

All the electrochemical measurements were carried out using an electrochemical workstation (CHI 760E, CHI Instrument, Shanghai, China) at room temperature in a conventional three-electrode cell with a Pt foil as the counter electrode and a Ag/AgCl electrode (with saturated KCl) as the reference electrode. The supporting electrolyte was saturated with high purity N_2_ for at least 20 min prior to each experiment and a N_2_ environment was kept over the solution in the cell. 

#### Material Characterization

The X-ray diffraction (XRD) data were obtained using a D8 Advance X-ray diffractometer (Cu Kα radiation λ = 0.1541 nm) operating at 40 kV and 25 mA. Field emission scanning electron microscopy (FESEM) images and energy-dispersive X-ray spectroscopy (EDS) analysis were recorded with a Gemini 300 instrument (Zeiss, Oberkochen, Germany). Fourier transform infrared spectroscopy (FT-IR) spectra were analyzed with a MAGNA-IR 750 spectrometer (Nicolet, Madison, WI, USA). The N_2_ adsorption-desorption measurement was executed on an Automated Gas Sorption Anlyzer (Quantachrome, Boynton Beach, FL, USA). Raman spectra were collected by a LabRAM HR Evolution Raman spectrometer with a 532 nm laser (HORIBA Scientific, Palaiseau, France). Transmission electron microscopy (TEM) and high resolution transmission electron microscopy (HRTEM) were performed on a Tecnai G2 F20 microscope (FEI, Hillsboro, OR, USA). The X-ray photoelectron spectroscopy (XPS) was carried on a K-Alpha spectrometer (Thermo Scientific, Waltham, MA, USA).

## 3. Results and Discussion

### 3.1. Structural Characterization

A well-defined columnar shape appeared in FeOOH-GA _0.125_, FeOOH-GA _0.25_ and FeOOH-GA _1.0_ (Figure S1, in SI). GO sheets were reduced by Fe^2+^ ion to form the aerogel. The volume of aerogel declined with Fe^2+^ ion increase. The FeOOH-GP takes the form of a black powder. 

**Figure 1:**
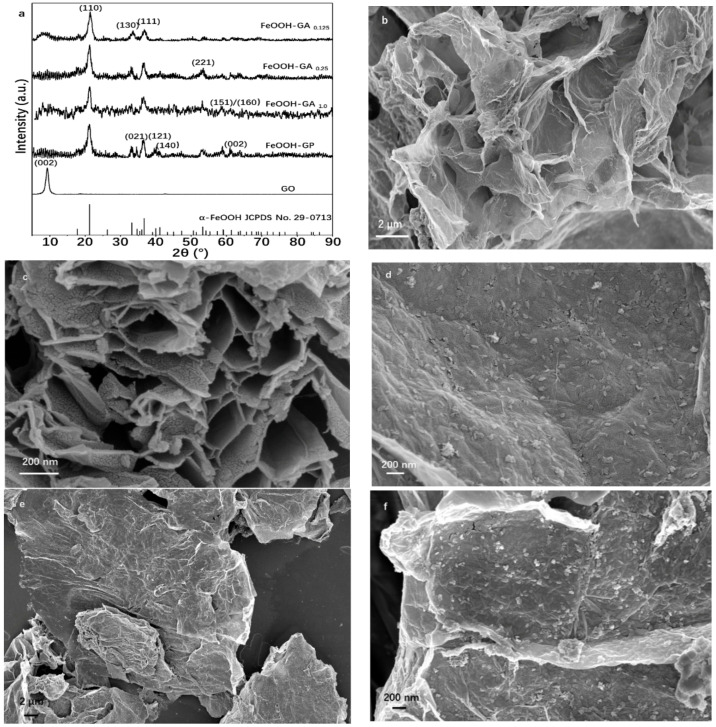
The XRD spectra of FeOOH-GA _0.125_, FeOOH-GA _0.25_, FeOOH-GA _1.0_, FeOOH-GP and GO (**a**). The typical FESEM images of FeOOH-GA _0.25_ (**b**–**d**) and FeOOH-GP (**e**,**f**).

X-ray powder diffraction (XRD) was used to investigate the crystallographic structures of FeOOH nanorods on reduced graphene oxide sheets for all samples. As illustrated in Figure 1a, the XRD pattern of GO has an obvious peak at 9.28° corresponding to the (002) plane of graphite [20,21]. However, the obvious peak at 9.28° disappeared in XRD patterns of all FeOOH-GA samples and FeOOH-GP. Meanwhile, there are new diffraction peaks in XRD patterns of all FeOOH-GA samples and FeOOH-GP, which are identified as α-FeOOH (JCPDS card No. 29-0713, orthorhombic system). As a result, a certain amount of α-FeOOH nanorods are anchored onto reduced graphene oxide sheets in all FeOOH-GA samples and FeOOH-GP. 

**Figure 2:**
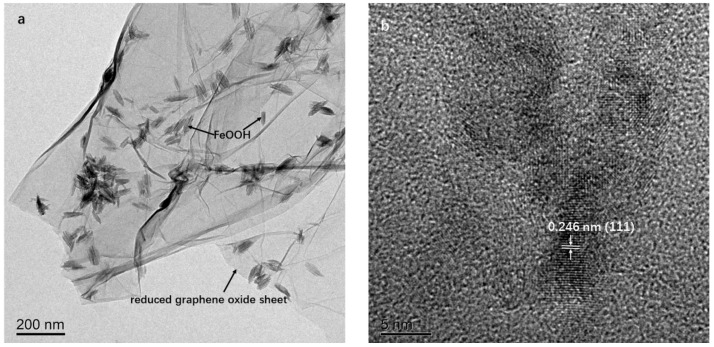
The typical TEM image of FeOOH-GA _0.25_ (**a**). The HRTEM image of FeOOH-GA _0.25_ (**b**).

The morphology and structure of the synthesized samples were investigated by FESEM and TEM. The typical FESEM images of FeOOH-GA _0.25_ (Figure 1b–d) exhibit obviously interconnected micro-sized pores and a mass of FeOOH nanorods with excellent dispersity on reduced graphene oxide sheets. The absence of micro-sized pores and the agglomerates of reduced graphene oxide sheets with FeOOH nanorods have been observed in FeOOH-GP (Figure 1e,f). The TEM image of FeOOH-GA _0.25_ (Figure 2a) also confirms that FeOOH nanorods load on the reduced graphene oxide sheets. Furthermore, the HETEM image of FeOOH-GA _0.25_ (Figure 2b) shows that the lattice distance of FeOOH nanorods is 0.246 nm, corresponding to the (111) plane of α-FeOOH, which is consistent with XRD data.

**Figure 3:**
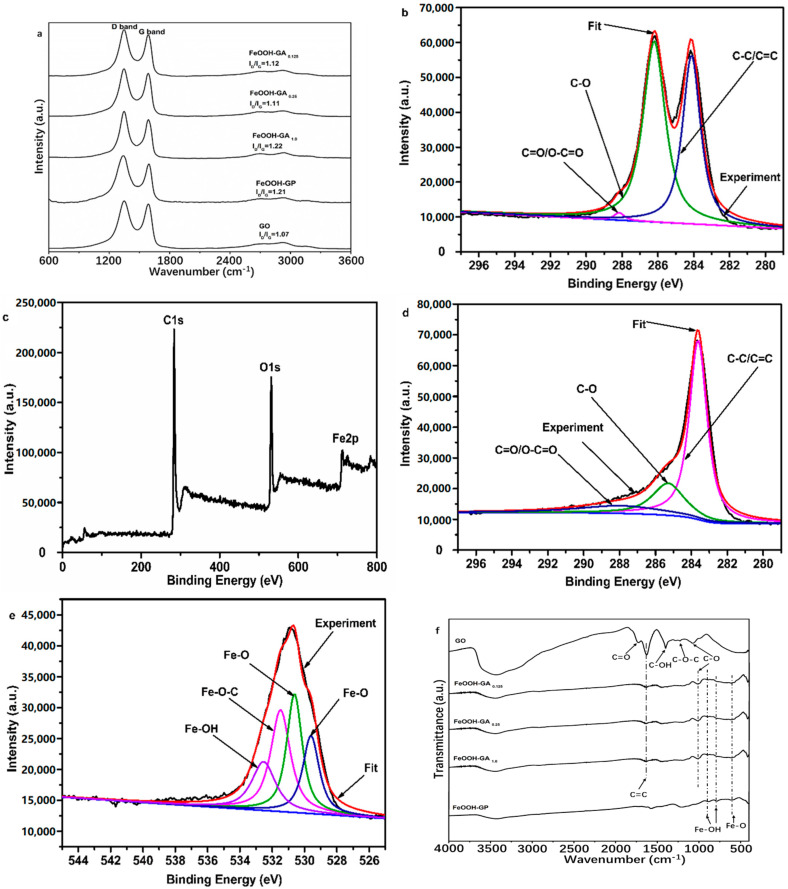
The Raman spectra of GO, FeOOH-GA _0.125_, FeOOH-GA _0.25_, FeOOH-GA _1.0_ and FeOOH-GP (**a**). The High resolution XPS spectrum of C 1s of GO (**b**). The full XPS spectrum of FeOOH-GA _0.25_ (**c**). The High resolution XPS spectra of C 1s (**d**) and O 1s (**e**) of FeOOH-GA _0.25_. The FT-IR spectra of GO, FeOOH-GA _0.125_, FeOOH-GA _0.25_, FeOOH-GA _1.0_ and FeOOH-GP (**f**).

A Raman spectrometer was used to reflect the quality of carbon materials. As shown in Figure 3a, Raman spectra of all samples reveal typical D and G bands. The intensity ratios of the D band and G band (I_D_/I_G_) in all FeOOH-GA samples and FeOOH-GP were increased compared to that of GO due to the presence of -FeOOH nanorods strengthening disorders and defects in the carbon skeleton [22].

Moreover, XPS, FT-IR, and EDS were further employed to probe the functional groups and the elemental composition of FeOOH-GA. The high-resolution C 1s spectrum of GO (Figure 3b) shows three peaks corresponding to C-C/C=C (284.1 eV), C-O (286.2 eV), and C=O/O-C=O (288.2 eV), respectively [23]. The element C 1s, O 1s, and Fe 2p peaks are exhibited in the full XPS spectrum of FeOOH-GA _0.25_ (Figure 3c) at 284 eV, 531 eV, and 711 eV, respectively. The peak intensity of C-O and C=O/O-C=O in the high-resolution C 1s spectrum of FeOOH-GA _0.25_ (Figure 3d) is significantly weaker than that of GO, indicating the oxygen-containing group reduced by Fe^2+^ ion [19,24,25]. The peaks at 724.6 eV (Fe 2p_1/2_) and 710.8 eV (Fe 2p_3/2_) are recognized as the characteristic peaks of α-FeOOH in the high-resolution Fe 2p spectrum of FeOOH-GA _0.25_ (Figure S2a) [19,26]. As shown in Figure 3e, the high resolution O 1s spectrum of FeOOH-GA _0.25_ can be deconvoluted into four peaks, which are typical of Fe-O (529.6 eV and 530.6 eV), Fe-O-C (531.5 eV), and Fe-OH (532.6 eV) [25,26]. The α-FeOOH nanorods anchor on reduced graphene oxide sheet through the Fe-O-C bond. The synergistic effects between the reduced graphene oxide sheet and α-FeOOH also pass the Fe-O-C bond [27]. 

As illustrated in Figure 3f, a great many oxygen-containing groups are observed from the FT-IR spectra of GO, which are attributed to carboxyl C=O stretching vibration (1737 cm^−1^), C=C vibration from the skeleton of GO sheets (1644 cm^−1^), C-OH vibration (1398 cm^−1^), C-O-C stretching vibration (1228 cm^−1^), and C-O stretching vibration (1079 cm^−1^), respectively [28]. However, three new characteristic peaks associated with Fe-OH (894.8 cm^−1^ and 784 cm^−1^) and Fe-O (602.7 cm^−1^) appear in FeOOH-GA _0.125_, FeOOH-GA _0.25_, FeOOH-GA _1.0_, and FeOOH-GP, which further confirmed the presence of FeOOH nanorods [19]. Meanwhile, the absorption peaks at 1737 cm^−1^, 1398 cm^−1^, 1228 cm^−1^, and 1079 cm^−1^ disappeared or clearly decreased. According to the above data, GO sheets were effectively reduced by Fe^2+^ ion to form reduced graphene oxide aerogel with FeOOH nanorods.

EDS patterns confirm the presence of elements C, O, Fe, and S in FeOOH-GA _0.125_ (Figure S2b), FeOOH-GA _0.25_ (Figure S2c), FeOOH-GA _1.0_ (Figure S2d), and FeOOH-GP (Figure S2e). Amongst them, the element Fe and the element O display strong signals. All Fe signals come from FeOOH nanorods. The atom content of Fe element is 3.82% in FeOOH-GA _0.125_, 6.39% in FeOOH-GA _0.25_, 10.98% in FeOOH-GA _1.0_, and 6.46% in FeOOH-GP, respectively, increasing with the elevation of the FeSO_4_·7H_2_O mass, indicating the content of FeOOH nanorods on reduced graphene oxide sheets. These values are near the atom content of Fe element in FeOOH-GA _0.25_ and FeOOH-GP prepared by reductant with the same mass. According to FT-IR and XPS data, oxygen-containing groups have been reduced by Fe^2+^ ion. The O singles are due to the existence of FeOOH nanorods. The single peak of S element comes from the residual sulfate radical. 

**Figure 4:**
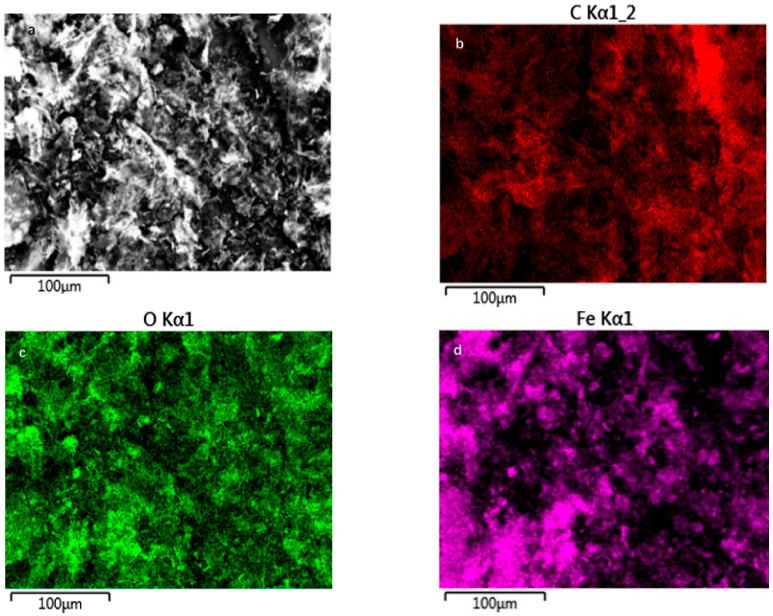

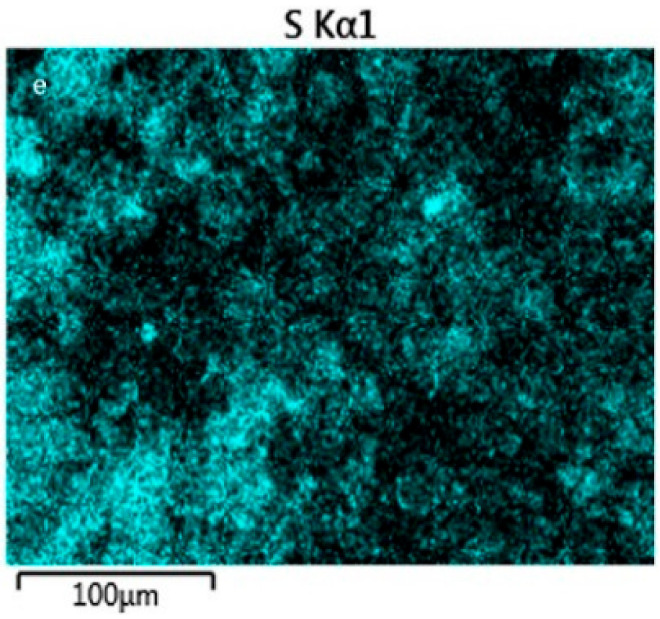
The FESEM image of FeOOH-GA _0.25_ (**a**) and corresponding element mapping images of C (**b**), O (**c**), Fe (**d**) and S (**e**).

EDS mapping was employed to confirm the distribution of elements in FeOOH-GA _0.25_. Figure 4b–e demonstrate the EDS elemental mapping analysis with a view to Figure 4a. The elemental analysis of FeOOH-GA _0.25_ clearly reveals the uniform distribution of C, O, Fe, and S elements on reduced graphene oxide sheets, demonstrating that FeOOH nanorods uniformly anchored on the reduced graphene oxide sheet.

**Figure 5:**
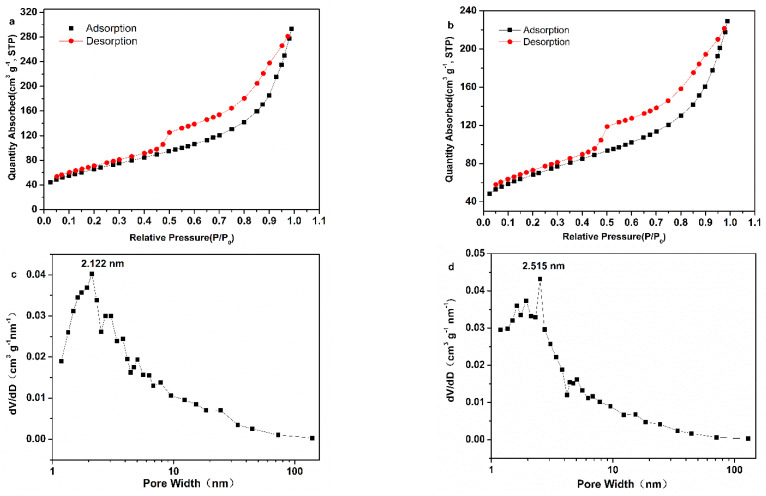
N_2_ sorption isotherms of FeOOH-GA _0.25_ (**a**) and FeOOH-GP (**b**), the pore size distribution from the BJH method of FeOOH-GA _0.25_ (**c**) and FeOOH-GP (**d**).

The porous structure of FeOOH-GA _0.25_ and FeOOH-GP were further confirmed by N_2_ adsorption-desorption measurement (<100 nm). The specific surface areas of FeOOH-GA _0.25_ and FeOOH-GP are 239.4 m^2^ g^−1^ and 237.6 m^2^ g^−1^, respectively (Figure 5a,b). The results of pore size distribution show a lot of mesopore from 2 to 10 nm in FeOOH-GA _0.25_ and FeOOH-GP. Then, the preponderant pore sizes are 2.122 and 2.515 nm, respectively (Figure 5c,d). The same amount of reducing agent brings about near specific surface area and pore size distribution. However, in light of the FESEM data, FeOOH-GA _0.25_ has a lot of micro-sized pores (>100 nm), which do not provide the specific surface areas, and the pores are mainly less than 100 nm in FeOOH-GP. Further, there are various size pores in FeOOH-GA, including micro-sized pores for the substrate solution swap and mesopores for enzyme immobilization.

### 3.2. Electrochemical Properties

**Figure 6:**
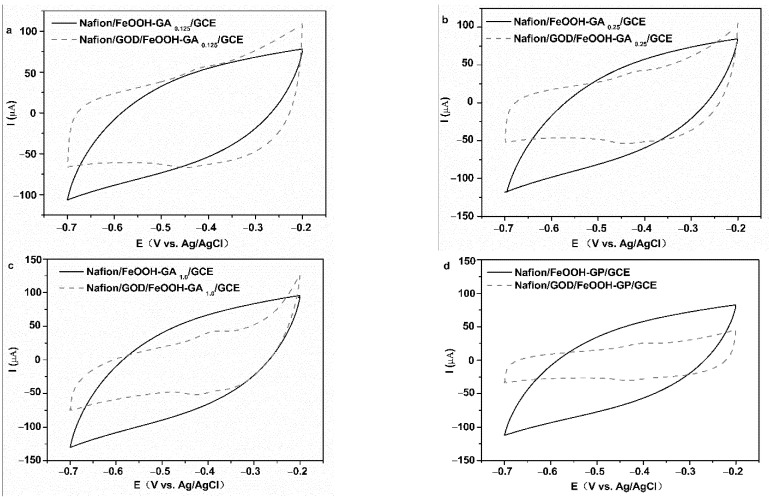
The CV curves of Nafion/FeOOH-GA _0.125_/GCE and Nafion/GOD/FeOOH-GA _0.125_/GCE (**a**), Nafion/FeOOH-GA _0.25_/GCE and Nafion/GOD/FeOOH-GA _0.25_/GCE (**b**), Nafion/FeOOH-GA _1.0_/GCE and Nafion/GOD/FeOOH-GA _1.0_/GCE (**c**), Nafion/FeOOH-GP/GCE and Nafion/GOD/FeOOH-GP/GCE (**d**) at 50 mV s^−1^ scan rate.

Cyclic voltammetry was employed to characterize the two types of electrodes in 0.2 M PBS (pH = 7). There are no well-defined redox peaks in curves of the Nafion/FeOOH-GA _0.125_/GCE, Nafion/FeOOH-GA _0.25_/GCE, Nafion/FeOOH-GA _1.0_/GCE, and Nafion/FeOOH-GP/GCE (Figure 6a–d). However, a pair of obvious redox peaks are displayed in the curves of Nafion/GOD/FeOOH-GA _0.125_/GCE, Nafion/GOD/FeOOH-GA _0.25_/GCE, Nafion/GOD/FeOOH-GA _1.0_/GCE, and Nafion/GOD/FeOOH-GP/GCE (Figure 6a–d).

The values of the anodic peak current (I_pa_) and cathodic peak current (I_pc_) in pair are approximately equal, decreasing with the increasing FeOOH quality. The peak currents of Nafion/GOD/FeOOH-GP/GCE are minimum because of the lack of porous structure. The formal potential (E^0′^) was calculated by the arithmetic mean value of the anodic peak potential (E_pa_) and cathodic peak potential (E_pc_). The potential difference (ΔE_p_) was calculated from E_pa_ and E_pc_. E^0′^ is positively shifted with increasing FeOOH quality (Table S1). The results indicate that the quasi-reversible redox reaction occurs on GOD modified electrodes and redox peaks are caused by GOD, which signifies that GOD has been successfully adsorbed on the carrier.

**Figure 7:**
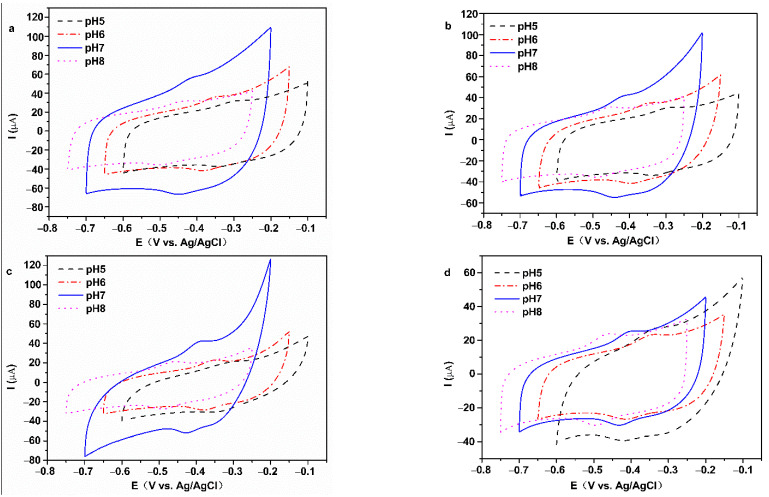
The CV curves of Nafion/GOD/FeOOH-GA _0.125_/GCE (**a**), Nafion/GOD/FeOOH-GA _0.25_/GCE (**b**), Nafion/GOD/FeOOH-GA _1.0_/GCE (**c**) and Nafion/GOD/FeOOH-GP/GCE (**d**) in various pH values of electrolyte with 50 mV s^−1^ scan rate.

The pH of phosphate buffer solution affects the electrochemical behavior of GOD in GOD modified electrodes. As shown in Figure 7a–d, CV curves of all GOD modified electrodes express obvious redox peaks from pH 5.0 to 8.0, and the peak currents of all GOD modified electrodes are the maximum at pH 7.0. The E_pa_ and E_pc_ shift to the negative direction when buffer solution pH is increased, which demonstrates that the electrochemical reaction of GOD on the electrode involves proton exchange.

The values of E^0′^ corresponding to working electrodes in Figure 7a–d present linear correlation curves with the pH of PBS (Figure S3). The slopes of Nafion/GOD/FeOOH-GA _0.125_/GCE, Nafion/GOD/FeOOH-GA _0.25_/GCE, and Nafion/GOD/FeOOH-GA _1.0_/GCE are −51.95 mV/pH (R^2^ = 0.9720), −51.10 mV/pH (R^2^ = 0.9906), and −50.35 mV/pH (R^2^ = 0.9865), respectively, closing to the theoretical value of −59.2 mV/pH for two-proton-transfer coupled and two-electron-transfer reaction [29,30]. These results prove that GOD on the electrode modified by FeOOH-GA displays a two-proton and two-electron transfer quasi-reversible electrochemical reaction, as represented by reaction (1) [31]:

GOD (FADH_2_) = GOD (FAD) + 2H^+^ + 2e^−^ (1)

The slope of Nafion/GOD/FeOOH-GP/GCE is −34.85 mV/pH, close to half of −59.2 mV/pH, which explains that the electrochemical reaction of GOD on the electrode modified by FeOOH-GP involves a two-proton and one-electron transfer [32]. The electrodes modified by FeOOH-GA with three-dimensional structure are more favourable to the electron transfer of GOD than electrodes modified by FeOOH-GP.

**Figure 8:**
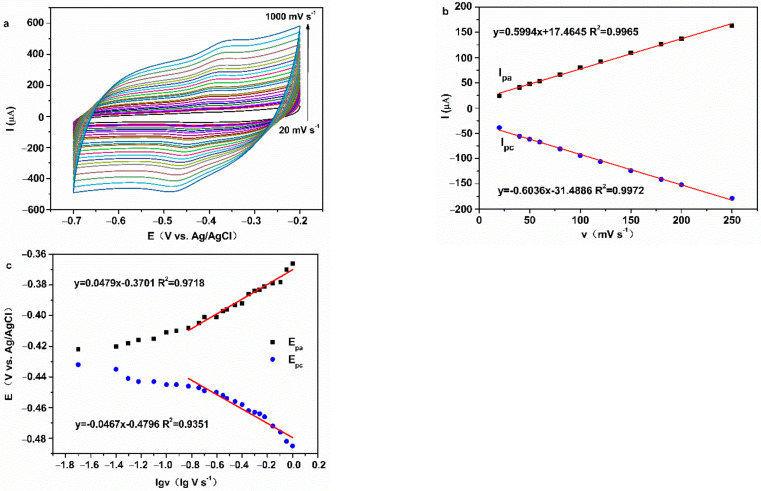
The CV curves of Nafion/GOD/FeOOH-GA _0.25_/GCE at various scan speed (**a**), polt of I_pa_ and I_pc_ vs. v for Nafion/GOD/FeOOH-GA _0.25_/GCE (**b**), polt of E_pa_ and E_pc_ vs. lgv for Nafion/GOD/FeOOH-GA _0.25_/GCE (**c**).

CV curves of all GOD electrodes in N_2_-saturated 0.2 M PBS (pH = 7.0) with different scan rates are shown in Figure 8 and Figures S4–S6. I_pa_ and I_pc_ of all GOD electrodes increased gradually with increasing scan rate and had a straight-line relationship with the scan rate from 20 to 250 mV s^−1^, accompanied by E_pa_ shifting to positive and E_pc_ shifting to negative.

Peak potentials had a straight-line relationship with the differential coefficient of scan rate, when the scan rate is greater than 150 mV s^−1^. These results indicate that all GOD electrodes reveal quasi-reversible, surface-controlled electrochemical process [31,33]. The kinetic parameter was calculated by Equations (2)–(4) [34,35,36]:

(2) $$E_{\mathrm{pa}}={E^{0}}^{\prime }+[\frac{2.3\mathrm{RT}}{\alpha \mathrm{nF}}]\mathrm{lg}[\frac{\alpha \mathrm{nF}}{{\mathrm{RTK}}_{s}}v]$$

(3) $$E_{\mathrm{pc}}={E^{0}}^{\prime }-[\frac{2.3\mathrm{RT}}{\left(1-\alpha \right)\mathrm{nF}}]\mathrm{lg}[\frac{\left(1-\alpha \right)\mathrm{nF}}{{\mathrm{RTK}}_{s}}v]$$

(4) $$\mathrm{lg}=\alpha \mathrm{lg}+\mathrm{lg}\alpha -\mathrm{lg}\frac{\mathrm{RT}}{\mathrm{nFv}}-\alpha \left(1-\alpha \right)(\frac{\mathrm{nF}\Delta E_{p}}{2.3\mathrm{RT}})$$

where α is the transfer coefficient, n is the number of electrons transferred, and K_s_ is the heterogeneous electron transfer rate constant. v, R, T, and F are the scan rate, the gas constant (8.314 J K^−1^ mol^−1^), ambient temperature (298 K), and Faraday constant (96,480 C mol^−1^), respectively. When the scan rate was 200 mV s^−1^, the K_s_ values of Nafion/GOD/FeOOH-GA _0.125_/GCE, Nafion/GOD/FeOOH-GA _0.25_/GCE, Nafion/GOD/FeOOH-GA _1.0_/GCE, and Nafion/GOD/FeOOH-GP/GCE are 2.457 s^−1^, 3.033 s^−1^, 1.683 s^−1^, and 2.449 s^−1^, respectively. These data demonstrate that the electron transfer rate is impeded by an excess amount of FeOOH and promoted by moderate FeOOH. In addition, the GOD electrode decorated by FeOOH-GA _0.25_ with porous structure offers faster interfacial electron transfer than the GOD electrode prepared by FeOOH-GP at a similar amount of FeOOH.

**Figure 9:**
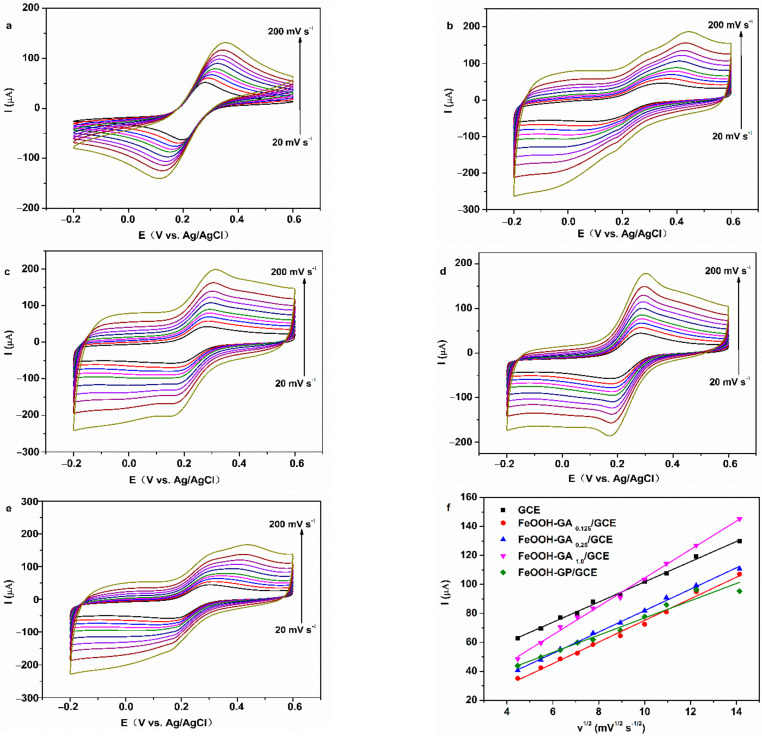
The CV curves of GCE (**a**), FeOOH-GA _0.125_/GCE (**b**), FeOOH-GA _0.25_/GCE (**c**), FeOOH-GA _1.0_/GCE (**d**) and FeOOH-GP/GCE (**e**) in 5 mM K_3_[Fe(CN)_6_] + 0.1 M KCl solution at various scan speed, (**f**) plo of I_pa_ vs. square root of scan rate from (**a**–**e**).

The effective surface areas were reflected through CV measurement in 5.0 mM K_3_[Fe(CN)_6_] + 0.1 M KCl solution with different scan rates from 20 to 200 mV s^−1^ for bare electrode and electrodes embellished with different carriers. The redox peaks demonstrate increasingly perfect symmetry with the increasing content of FeOOH (Figure 9b–d), and the symmetry of FeOOH-GA _0.25_/GCE is better than that of FeOOH-GP/GCE (Figure 9e). In addition, a linear relationship was presented between ther square root of scan rate and the values of E_pc_ for the bare electrode and electrodes embellished with different carriers (Figure 9f). The values of I_pa_ for FeOOH-GA _0.25_/GCE are higher than that for FeOOH-GP/GCE at scan rate greater than 50 mV s^−1^, because the abundant macroporous structure in FeOOH-GA _0.25_ is beneficial to electrolyte diffusion and electron transfer.

Moreover, the effective surface areas were obtained from the Randles–Sevcik Equation (5) [37]:

(5) Ip=0.4463× nFAC(nFvDRT)12

where I_p_ is the peak current, A is the effective surface area, C is the bulk concentration (5 × 10^−6^ mol cm^−3^), D is the diffusion coefficient (7.6 × 10^−6^ cm^3^ s^−1^), and n (n = 1), v, F, R, and T are the same as in Equations (2)–(4).

Electrochemical data concerning the bare electrode and electrodes embellished with different carriers are shown in Table S2 about Figure 9. The effective surface areas of all FeOOH-GA embellished electrodes are higher than that of bare electrode (0.0930 cm^2^). However, the effective surface area of the FeOOH-GP (0.0793 cm^2^) embellished electrode is lower than that of bare electrode. At 50 mV s^−1^ scan rate, the values of ΔE_p_ for FeOOH-GA embellished electrodes decreased as the content of FeOOH increased. The values of ΔE_p_ for FeOOH-GA _0.25_/GCE (116 mV) and FeOOH-GA _1.0_/GCE (98 mv) are lower than that for the bare electrode (144 mV), which declares that the reversibility of electrodes decorated by FeOOH-GA _0.25_ and FeOOH-GA _1.0_ is better than that of the bare electrode. Meanwhile, the value of ΔE_p_ for FeOOH-GP/GCE (193 mV) is higher than that for FeOOH-GA _0.25_/GCE and bare electrode, which certifies that because of a lacking macroporous structure, the reversibility of FeOOH-GP/GCE is inferior to that of FeOOH-GA _0.25_/GCE and the bare electrode [38].

**Figure 10:**
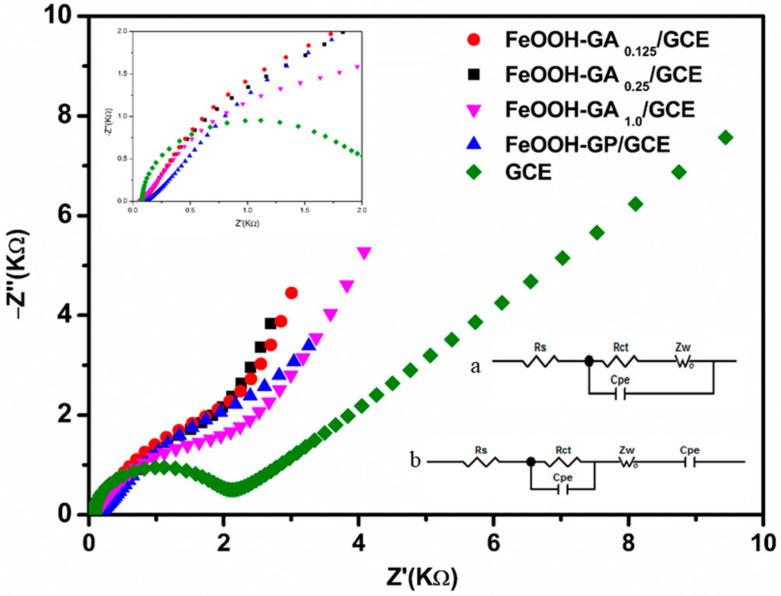
Electrochemical impedance spectra of GCE, FeOOH-GA _0.125_/GCE, FeOOH-GA _0.25_/GCE, FeOOH-GA _1.0_/GCE and FeOOH-GP/GCE. The right inset is the equivalent circuit of GCE (a) and FeOOH-GA _0.125_/GCE, FeOOH-GA _0.25_/GCE, FeOOH-GA _1.0_/GCE and FeOOH-GP/GCE (b). The left inset is the Nyquist polts of all working electrodes in the high frequency region).

Impedance information concerning electrodes modified by FeOOH-GA and FeOOH-GP was provided by electrochemical impedance spectroscopy (EIS). The equivalent circuit is shown in the lower right corner of Figure 10. In the circuit, R_s_ is the electrolyte resistance and R_ct_ is the charge transfer resistance. The values of R_ct_ for FeOOH-GP/GCE, FeOOH-GA _0.125_/GCE, FeOOH-GA _0.25_/GCE, and FeOOH-GA _1.0_/GCE are 1173, 1429, 1178, and 1214 Ω, respectively. Simultaneously, all values of R_ct_ for modified electrodes are smaller than that for GCE (1806 Ω), which expounds that the wonderful conductivity of samples prepared from reduced graphene oxide effectively reduced the resistance of charge transfer. The value of R_ct_ also reflects the presence of fairly strong hydrophobicity in reduced graphene oxide-based samples, which explains that GOD is adsorbed to reduced graphene oxide sheet and internal pores through the hydrophobic effect [39].

The values of R_s_ for FeOOH-GA _0.125_/GCE (74.2 Ω), FeOOH-GA _0.25_/GCE (62.72 Ω), and FeOOH-GA _1.0_/GCE (57.47 Ω) are smaller than that of GCE (82.13 Ω). However, the value of R_s_ for FeOOH-GP/GCE is 115.4 Ω. All values of R_s_ demonstrate that FeOOH-GA with porous structure and moderate FeOOH is conducive to the diffusion of electrolyte, leading to sufficient contact between the electrolyte and the electrode, which decreases the resistance of the electrolyte.

### 3.3. Electrocatalytic Properties

**Figure 11:**
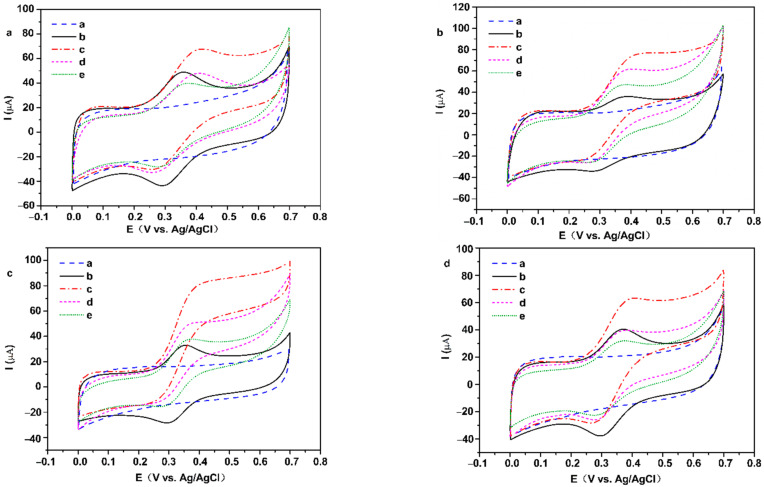

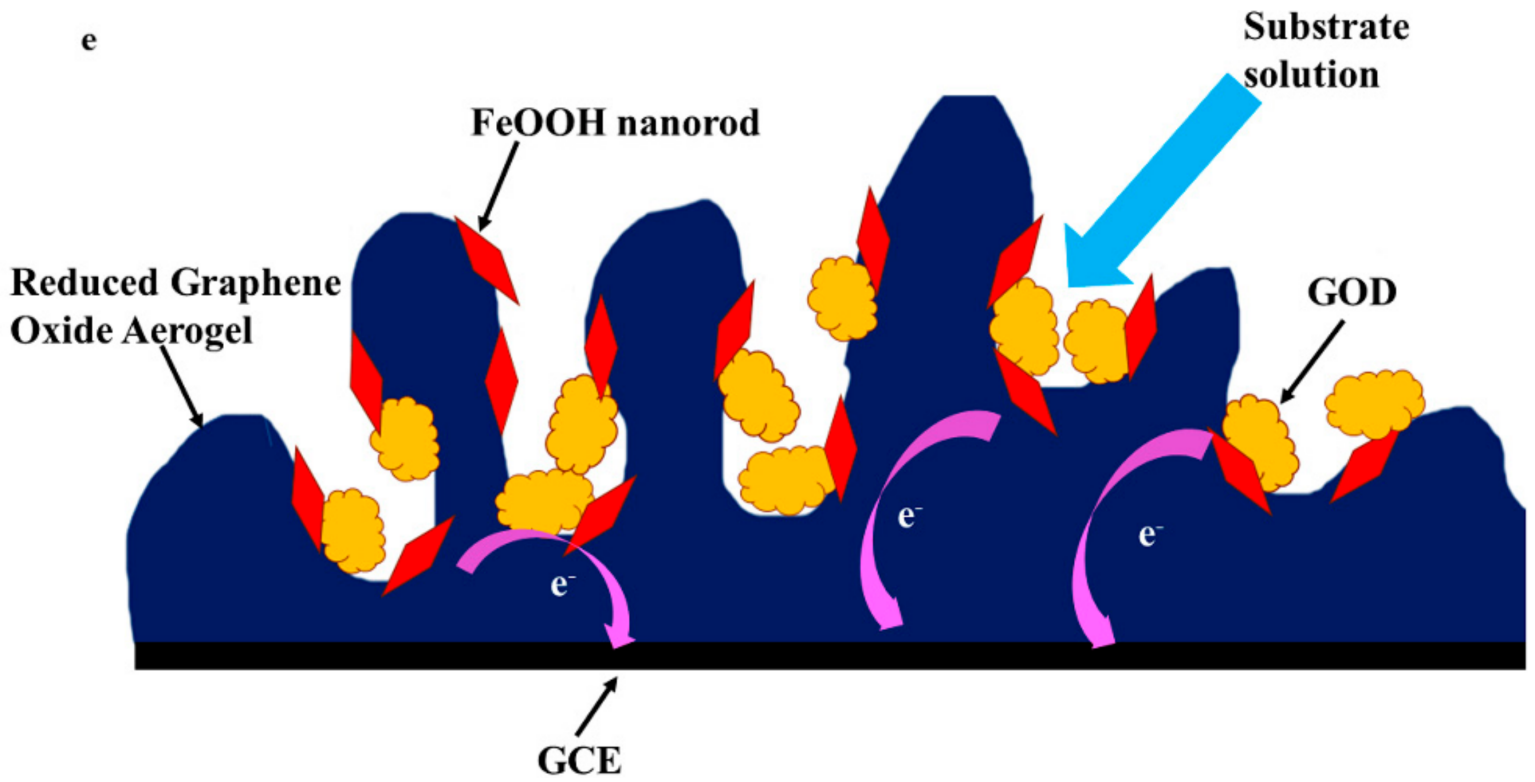
The CV curves of Nafion/GOD/FeOOH-GA _0.125_/GCE (**a**), Nafion/GOD/FeOOH-GA _0.25_/GCE (**b**), Nafion/GOD/FeOOH-GA _1.0_/GCE (**c**) and Nafion/GOD/FeOOH-GP/GCE (**d**) in PBS (pH = 7, curve a), PBS including 1 mM Fc (curve b), PBS including 1 mM Fc + 50 mM glucose (curve c at the first day, curve d after 7 days, curve e after 14 days) at 50 mV s^−1^ scan rate. The illustration of Nafion/FeOOH-GA/GCE for electrocatalysis (**e**).

To investigate the electrocatalytic properties of GOD on different working electrodes, CV tests were employed in PBS (pH = 7, containing 1mM Fc and 50 mM glucose) from 0.0 to 0.7 V (vs. Ag/AgCl) for different working electrodes (including Nafion/GOD/FeOOH-GA _0.125_/GCE, Nafion/GOD/FeOOH-GA _0.25_/GCE, Nafion/GOD/FeOOH-GA _1.0_/GCE, and Nafion/GOD/FeOOH-GP/GCE). All working electrodes do not emerge redox peaks in PBS (pH = 7, Figure 11 curves a). However, there are outstanding symmetrical redox peaks of Fc from all working electrodes in PBS (pH = 7, containing 1mM Fc, Figure 11 curves b). In addition, oxidation peak currents of all working electrodes are enhanced in PBS (pH = 7, containing 1 mM Fc and 50 mM glucose, Figure 11 curves c), which testifies that GOD immobilized on working electrodes can catalyze the oxidation of glucose and maintain bioelectrocatalytic activity. The oxidation peak current of glucose from Nafion/GOD/FeOOH-GA _1.0_/GCE is the highest among all working electrodes, expounding a mass of enzymes immobilized by OH^-^ of FeOOH nanorods. The oxidation peak current of glucose from Nafion/GOD/FeOOH-GA _0.25_/GCE is stronger than that of glucose from Nafion/GOD/FeOOH-GP/GCE as a result of the macroporous structure in FeOOH-GA _0.25_ that adsorbed more enzymes than FeOOH-GP and supplied a pathway to the substrate solution.

All working electrodes were placed in 0.2 M PBS (pH = 7) at 4 °C in a refrigerator and changed solution every three days. The oxidation peak current of glucose from Nafion/GOD/FeOOH-GA _0.25_/GCE still keeps 81.11% of the original current after seven days (Figure 11b, curve d). However, the oxidation peak current of glucose on Nafion/GOD/FeOOH-GA _1.0_/GCE only keeps 61.24% of the original current after seven days (Figure 11c, curve d), because poorly adsorbed enzymes are leaked and the construction of the FeOOH nanorod is collapsed as the storage time is prolonged. The heights of oxidation peaks from Nafion/GOD/FeOOH-GP/GCE and Nafion/GOD/FeOOH-GA _0.125_/GCE are weaker than that of Fc after 14 days (Figure 11a,d, curves e). Simultaneously, the value of the oxidation peak current from glucose on Nafion/GOD/FeOOH-GA _0.25_/GCE still keeps 61.84% of its initial value (Figure 11b, curve e), which indicates that a porous structure and suitable FeOOH content effectively prevent the loss of GOD and maintain the catalytic activity of GOD. 

To investigate the repeatability of different working electrodes, each type GOD electrodes was made into four electrodes for the CV test in PBS (pH = 7, containing 1mM Fc and 50 mM glucose). The values of relative standard deviation (RSD) in Nafion/GOD/FeOOH-GA _0.125_/GCE, Nafion/GOD/FeOOH-GA _0.25_/GCE, Nafion/GOD/FeOOH-GA _1.0_/GCE, and Nafion/GOD/FeOOH-GP/GCE are 3.7%, 4.7%, 6.4%, and 5.79%, respectively, which confirms different working electrodes with reasonable reproducibility. 

A cyclic voltammeter was also employed to determine the electrocatalytic properties of FeOOH-GA _0.25._ As shown in Figure S7, redox peaks did note increase on the CV curve of Nafion/FeOOH-GA _0.25_/GCE in 0.2 M PBS (pH = 7 containing 50 mM glucose). Furthermore, a pair of well-defined redox peaks emerge on the CV curve of Nafion/FeOOH-GA _0.25_/GCE in 0.2 M PBS with Fc and glucose, connecting with Fc instead of glucose, which confirms that the oxidation of glucose is facilitated by GOD rather than FeOOH-GA _0.25_.

The schematic structure of Nafion/GOD/FeOOH-GA/GCE is illustrated in Figure 11e. FeOOH-GA with plentifully porous structure and strong hydrophobicity could adsorb a large quantity of GOD into interior pore channels. The substrate solution flowed in and out of pore channels and sufficiently contacted GOD, bringing about the oxidation reaction of glucose. Generated electrons could rapidly transfer to the electrode due to the excellent electrical conductivity of the reduced graphene oxide sheet. Meanwhile, a large number of FeOOH nanorods anchored on the reduced graphene oxide sheet could fix enzymes by hydroxyl groups (OH^−^) and form speed bumps, preventing the leave of enzyme on the working electrode, which was advantageous for the preservation of working electrodes. The low content of FeOOH could not supply effective resistance. Thus, an excessive content of FeOOH would cause structural collapse. This is also not beneficial to the preservation of GOD electrodes.

## 4. Conclusions

The α-FeOOH-reduced graphene oxide aerogel was successful synthesized using graphene oxide and FeSO_4_·7H_2_O. The results of Raman, XPS, XRD, FT-IR, EDS mapping, and TEM reveal that graphene oxide is reduced by Fe^2+^ ion and that α-FeOOH nanorods uniformly anchored on reduced graphene oxide sheet through the Fe-O-C bond. The α-FeOOH-reduced graphene oxide aerogel also has a various and interconnected pore structure through analyses utilizing SEM and the Brunauer–Emmett–Teller method. 

The α-FeOOH-reduced graphene oxide aerogel was used as a carrier for the immobilization of GOD, forming the working electrode. The electrochemical data display that GOD on the electrode modified by FeOOH-GA belongs to a two-proton and two-electron transfer quasi-reversible electrochemical reaction. FeOOH-GA could effectively immobilize enzymes because of the diversified pore structure, powerful hydrophobicity, and FeOOH nanorods. The GOD immobilized on working electrodes maintain bioelectrocatalytic activity. Furthermore, the porosity of the α-FeOOH-reduced graphene oxide aerogel allowed for the diffusion of electrolyte, sufficient contact between electrolyte and electrodes, and the diminution of resistance in the electrolyte. Appropriate α-FeOOH and diversified pore structure not only improved electron transfer and enzyme electrode storage time, but also offered a quick interfacial electron transfer rate. The value of K_s_ (3.033 s^−1^) in Nafion/GOD/FeOOH-GA _0.25_/GCE is higher than that in the GOD electrode prepared by FeOOH-GP without diversified pore structure. The GOD electrode modified by FeOOH-GA _0.25_ maintained 81.11% of original current after seven days and 61.84% of the initial value after 14 days, respectively. These results indicate that FeOOH-GA can be considered an excellent carrier used for enzyme immobilization.

## Data Availability

The data produced from this study can be requested from the corresponding author, upon reasonable request.

## Supplementary Materials

The following supporting information can be downloaded at: https://www.mdpi.com/article/10.3390/membranes12050447/s1, Figure S1: The photos of FeOOH-GA _0.125_ (a), FeOOH-GA _0.25_ (b), FeOOH-GA _1.0_ (c) and FeOOH-GP (d); Figure S2: The High resolution XPS spectrum of Fe 2p of FeOOH-GA _0.25_ (a). EDS spectra of FeOOH-GA _0.125_ (b), FeOOH-GA _0.25_ (c), FeOOH-GA _1.0_ (d) and FeOOH-GP (e); Figure S3: Plot of E_0′_ vs. pH value for Nafion/GOD/FeOOH-GA _0.125_/GCE (a), Nafion/GOD/FeOOH-GA _0.25_/GCE (b), Nafion/GOD/FeOOH-GA _1.0_/GCE (c) and Nafion/GOD/FeOOH-GP/GCE (d); Figure S4: CV curves of Nafion/GOD/FeOOH-GA _0.125_/GCE at various scan speed (a), polt of Ipa and Ipc vs. v for Nafion/GOD/FeOOH-GA _0.125_/GCE (b), polt of E_pa_ and E_pc_ vs. lgv for Nafion/GOD/FeOOH-GA _0.125_/GCE (c); Figure S5: CV curves of Nafion/GOD/FeOOH-GA _1.0_/GCE at various scan speed (a), polt of I_pa_ and I_pc_ vs. v for Nafion/GOD/FeOOH-GA _1.0_/GCE (b), polt of E_pa_ and E_pc_ vs. lgv for Nafion/GOD/FeOOH-GA _1.0_/GCE (c); Figure S6: CV curves of Nafion/GOD/FeOOH-GP/GCE at various scan speed (a), polt of Ipa and Ipc vs. v for Nafion/GOD/FeOOH-GP/GCE (b), polt of Epa and Epc vs. lgv for Nafion/GOD/FeOOH-GP/GCE (c); Figure S7: CV curves of Nafion/FeOOH-GA _0.25_/GCE in different solutions; Table S1: The potential values of different working electrodes; Table S2: Electrochemical data of different working electrodes.
