# Supplementary material for: Construct α-FeOOH-Reduced Graphene Oxide Aerogel as a Carrier for Glucose Oxidase Electrode
Source: Membranes (Basel). 2022 Apr 21;12(5):447. doi: 10.3390/membranes12050447 (PMC9146937; doi:10.3390/membranes12050447)
Supplement: Supplementary file 1 [file membranes-12-00447-s001.zip › membranes-1635561-supplementary.pdf]

membranes

MDPI

Supplementary Material for:

Construct α-FeOOH-Reduced Graphene Oxide Aerogel as a Car-Rier for Glucose Oxidase Electrode

Yue Yao [1,2,*], ChangYu Hou [1] and Xin Zhang [2]

[1] College of Chemistry and Materials Engineering, Anhui Science and Technology University, Bengbu, Anhui 233030, China
[2] Department of Chemistry, College of Science, Shantou University, Shantou 515063, China
* Correspondence: yaoyue@ahstu.edu.cn

Supplementary Information

Experimental Section

Graphene oxide (GO) was synthesized from natural graphite powder by a modified Hummer's method [1,2].

In a typical reaction, 25 mL H_2SO_4, 2.25 g P_2O_5 and 2.25 g $(HN_4)_2S_2O_8$ were stirred in a round-bottom flask under 80 °C oil bath, then added 2.5 g graphite (325 mesh), following stirred with reflux at 80 °C oil bath for 4.5 h. Then the round-bottom flask was taken out for cooling to indoor temperature, added deionized water and placed for 12 h. Solution was filtered by vacuum filtration with 0.45 μm filter membrane and washed with deionized water until the pH of supernatant turned to 7.0. The filter cake was dried at 60 °C.

Dried filter cake and 100 mL H_2SO_4 were stirred under ice bath. 12.5 g $KMnO_4$ was slowly added into solution and stirred for 1 h. The solution was transferred to 35 °C water bath and stirred for 4 h. Then the reaction solution was cooled to indoor temperature and slowly added 200 mL deionized water with stirring, following 35 °C water bath for 2 h. 625 mL deionized water was added at ice bath. 25 mL 30% H_2O_2 was added to remove the $KMnO_4$ and MnO_2. The mixture solution was placed for 12 h, removed the supernatant, successively centrifugally washed by 500 mL 10 % HCl and deionized water at 10000 r/min until the pH of solution near to neutral. GO was subjected to dialysis for one week to remove residual salts and acids. Finally, graphene oxide aqueous dispersion was stored at 4 °C.

References

1. Hummers, W.S.; Offenman, J.R.R.E. Preparation of Graphitic Oxide. *J. Am. Chem. Soc.* **1958**, *80*, 1339.
2. Ji, L.; Chen, W.; Xu, Z.; Zheng, S.; Zhu, D. Graphene Nanosheets and Graphite Oxide as Promising Adsorbents for Removal of Organic Contaminants from Aqueous Solution. *J. Environ. Qual.* **2013**, *42*, 191.

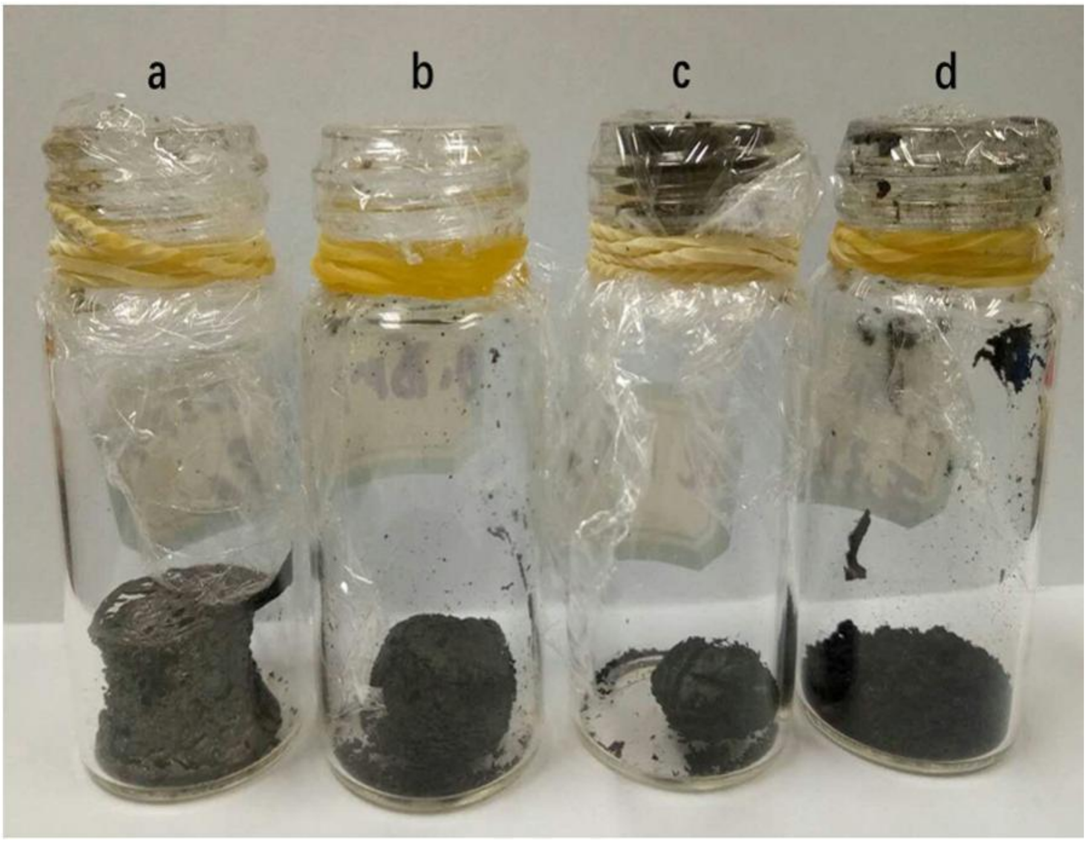

Figure S1. The photos of FeOOH-GA $_{0.125}$ (**a**), FeOOH-GA $_{0.25}$ (**b**), FeOOH-GA $_{1.0}$ (**c**) and FeOOH-GP (**d**).

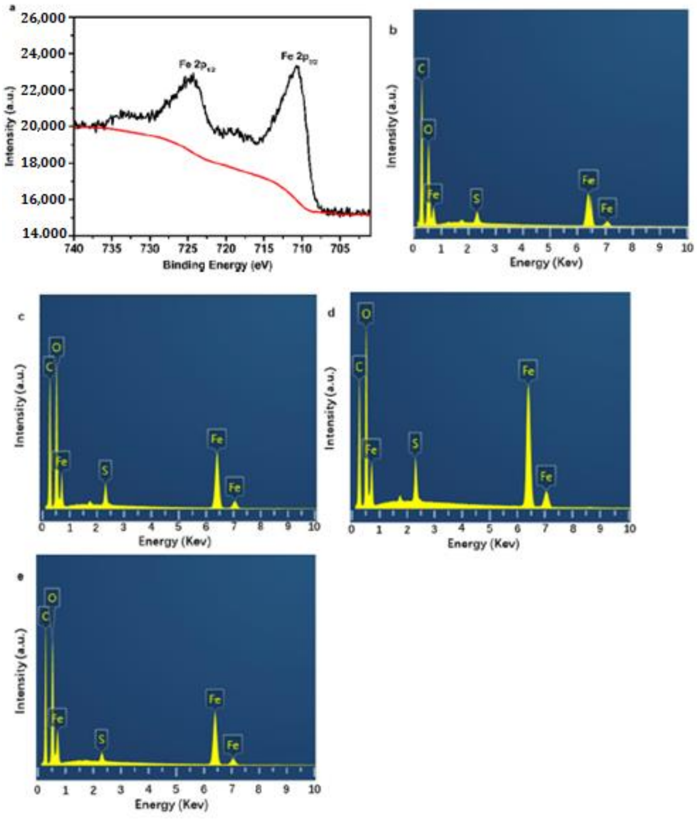

Figure S2. The High resolution XPS spectrum of Fe 2p of FeOOH-GA $_{0.25}$ (**a**). EDS spectra of FeOOH-GA $_{0.125}$ (**b**), FeOOH-GA $_{0.25}$ (**c**), FeOOH-GA $_{1.0}$ (**d**) and FeOOH-GP (**e**).

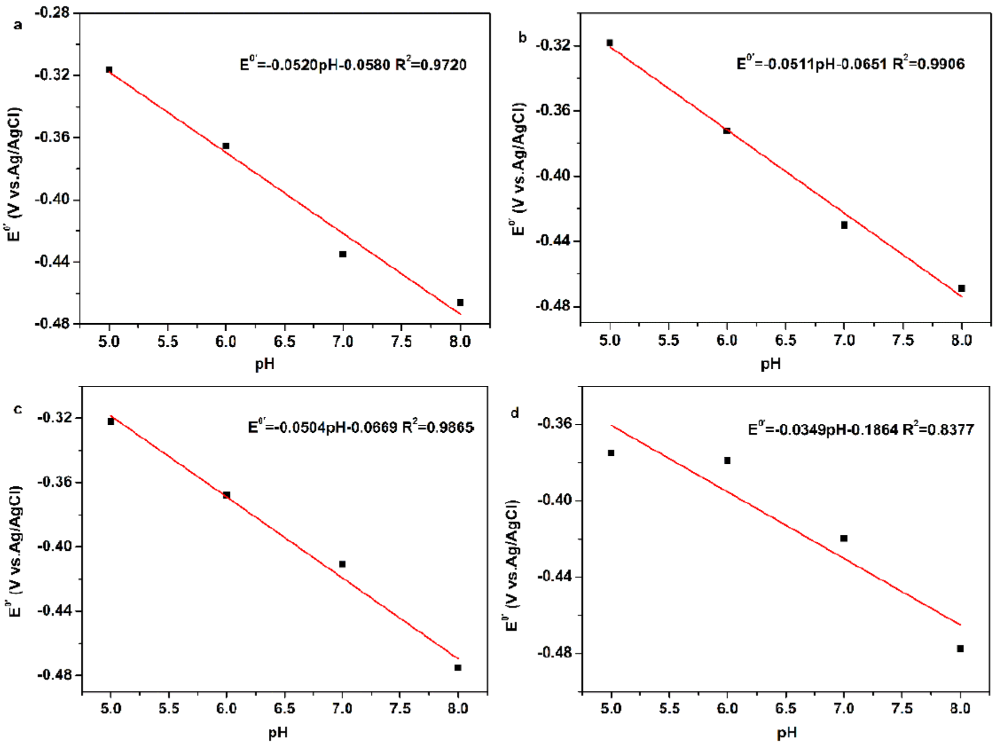

Figure S3. Plot of $E_{0'}$ vs. pH value for Nafion/GOD/FeOOH-GA $_{0.125}$/GCE (**a**), Nafion/GOD/FeOOH-GA $_{0.25}$/GCE (**b**), Nafion/GOD/FeOOH-GA $_{1.0}$/GCE (**c**) and Nafion/GOD/FeOOH-GP/GCE (**d**).

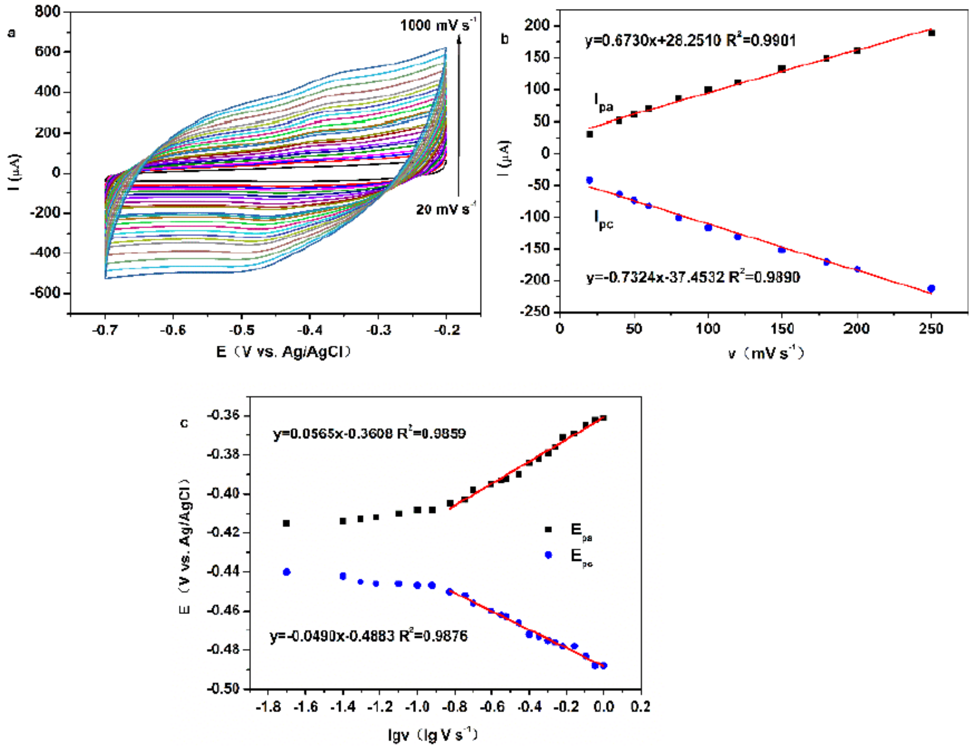

Figure S4. CV curves of Nafion/GOD/FeOOH-GA $_{0.125}$/GCE at various scan speed (**a**), polt of I_{pa} and I_{pc} vs. v for Nafion/GOD/FeOOH-GA $_{0.125}$/GCE (**b**), polt of E_{pa} and E_{pc} vs. lgv for Nafion/GOD/FeOOH-GA $_{0.125}$/GCE (**c**).

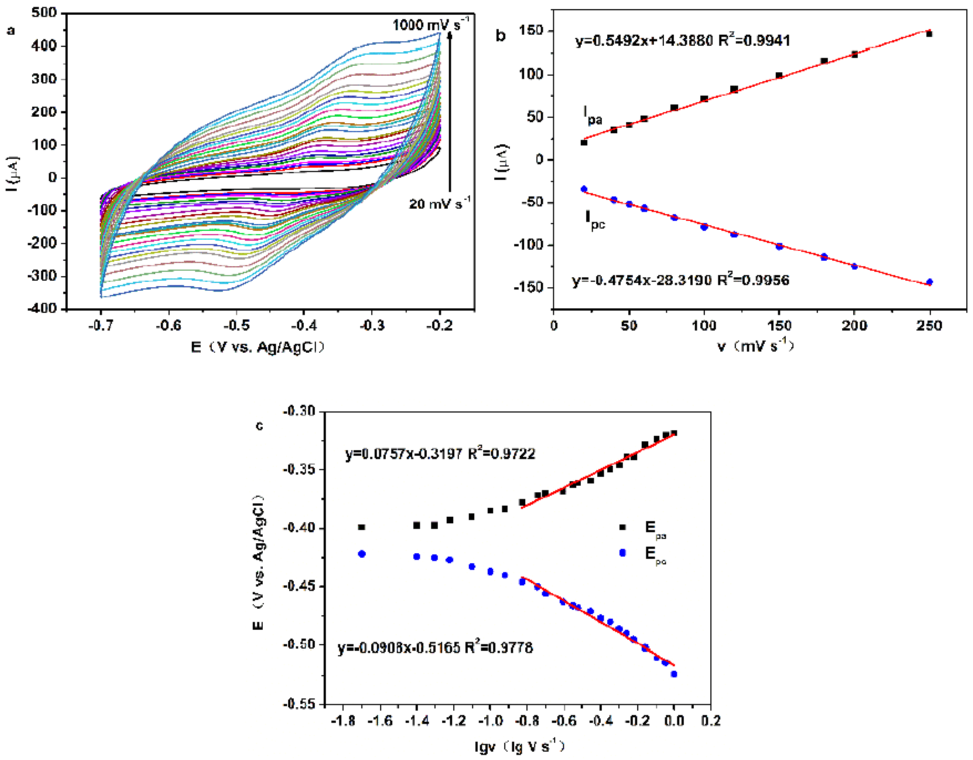

Figure S5. CV curves of Nafion/GOD/FeOOH-GA $_{1.0}$/GCE at various scan speed (**a**), polt of I_{pa} and I_{pc} vs. v for Nafion/GOD/FeOOH-GA $_{1.0}$/GCE (**b**), polt of E_{pa} and E_{pc} vs. lgv for Nafion/GOD/FeOOH-GA $_{1.0}$/GCE (**c**).

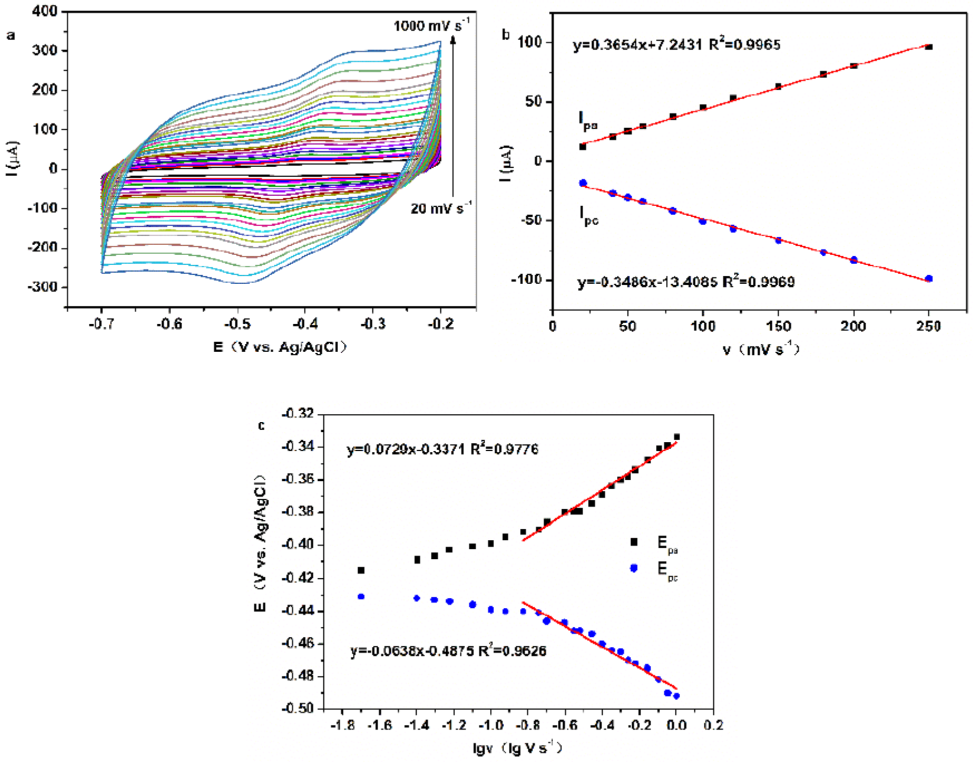

Figure S6. CV curves of Nafion/GOD/FeOOH-GP/GCE at various scan speed (**a**), polt of I_{pa} and I_{pc} vs. v for Nafion/GOD/FeOOH-GP/GCE (**b**), polt of E_{pa} and E_{pc} vs. lgv for Nafion/GOD/FeOOH-GP/GCE (**c**).

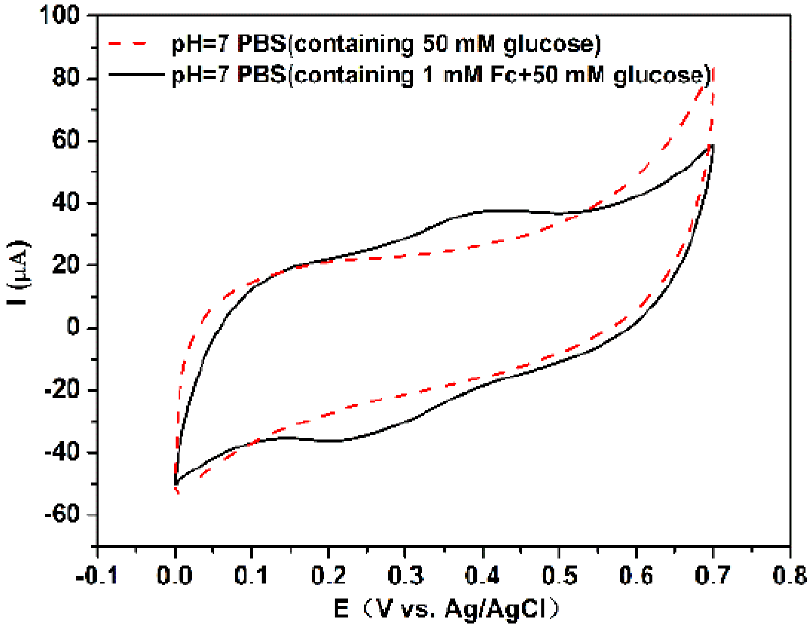

Figure S7. CV curves of Nafion/FeOOH-GA $_{0.25}$/GCE in different solutions.

Table S1. The potential values of different working electrodes.

Names of Electrodes	E_{pa}	E_{pc}	ΔE_p	$E^{0'}$
Nafion/GOD/FeOOH-GA $_{0.125}$/GCE	−0.420 V	−0.450 V	30 mV	−0.435 V
Nafion/GOD/FeOOH-GA $_{0.25}$/GCE	−0.415 V	−0.443 V	28 mV	−0.429 V
Nafion/GOD/FeOOH-GA $_{1.0}$/GCE	−0.397 V	−0.425 V	28 mV	−0.411 V
Nafion/GOD/FeOOH-GP/GCE	−0.407 V	−0.433 V	26 mV	−0.420 V

Table S2. Electrochemical data of different working electrodes.

Names of Electrodes	Fitted Curve	A (cm^2)	ΔE_p (50 mV s^{-1})
GCE	y = 6.9728x + 32.0440（R^2 = 0.9958）	0.0930	144 mV
FeOOH-GA $_{0.125}$/GCE	y = 7.4610x + 0.5374（R^2 = 0.9930）	0.0995	313 mV
FeOOH-GA $_{0.25}$/GCE	y = 7.3890x + 8.0342（R^2 = 0.9975）	0.0985	116 mV
FeOOH-GA $_{1.0}$/GCE	y = 9.8148x + 6.3129（R^2 = 0.9966）	0.1309	98 mV
FeOOH-GP/GCE	y = 5.9438x + 17.4210（R^2 = 0.9654）	0.0793	193 mV
